# The number and choice of muscles impact the results of muscle synergy analyses

**DOI:** 10.3389/fncom.2013.00105

**Published:** 2013-08-08

**Authors:** Katherine M. Steele, Matthew C. Tresch, Eric J. Perreault

**Affiliations:** ^1^Mechanical Engineering, University of WashingtonSeattle, WA, USA; ^2^Sensorimotor Performance Program, Rehabilitation Institute of ChicagoChicago, IL, USA; ^3^Biomedical Engineering, Northwestern UniversityEvanston, IL, USA; ^4^Department of Physical Medicine and Rehabilitation, Northwestern University Feinberg School of MedicineChicago, IL, USA

**Keywords:** muscle synergy, electromyography, simulation, nonnegative matrix factorization, musculoskeletal model

## Abstract

One theory for how humans control movement is that muscles are activated in weighted groups or synergies. Studies have shown that electromyography (EMG) from a variety of tasks can be described by a low-dimensional space thought to reflect synergies. These studies use algorithms, such as nonnegative matrix factorization, to identify synergies from EMG. Due to experimental constraints, EMG can rarely be taken from all muscles involved in a task. However, it is unclear if the choice of muscles included in the analysis impacts estimated synergies. The aim of our study was to evaluate the impact of the number and choice of muscles on synergy analyses. We used a musculoskeletal model to calculate muscle activations required to perform an isometric upper-extremity task. Synergies calculated from the activations from the musculoskeletal model were similar to a prior experimental study. To evaluate the impact of the number of muscles included in the analysis, we randomly selected subsets of between 5 and 29 muscles and compared the similarity of the synergies calculated from each subset to a master set of synergies calculated from all muscles. We determined that the structure of synergies is dependent upon the number and choice of muscles included in the analysis. When five muscles were included in the analysis, the similarity of the synergies to the master set was only 0.57 ± 0.54; however, the similarity improved to over 0.8 with more than ten muscles. We identified two methods, selecting dominant muscles from the master set or selecting muscles with the largest maximum isometric force, which significantly improved similarity to the master set and can help guide future experimental design. Analyses that included a small subset of muscles also over-estimated the variance accounted for (VAF) by the synergies compared to an analysis with all muscles. Thus, researchers should use caution using VAF to evaluate synergies when EMG is measured from a small subset of muscles.

## Introduction

The human musculoskeletal system is complex, providing a robust and flexible system for executing tasks of daily life. A primary challenge for researchers, clinicians, and others seeking to evaluate human movement is to understand how we control this complex system. The musculoskeletal system is highly redundant, with more muscles than degrees of freedom. Thus, there are many different ways that muscles can be recruited to execute a given task. Understanding the control strategies used during movement can provide insight into pathologic conditions, optimize performance, and inspire the design of novel robotics.

One theory of the control of human movement suggests that muscles are activated in groups, commonly referred to as synergies or modes (Lee, 1984; Tresch et al., 1999; Krishnamoorthy et al., 2003; Ting and Macpherson, 2005; Ivanenko et al., 2006). Activating multiple muscles with a single control signal is theorized to provide a simplified system compared to controlling each muscle individually. Previous studies have shown that muscle activity during a variety of tasks in humans (Ting and Macpherson, 2005; Ivanenko et al., 2007; Cheung et al., 2012) and animals (Tresch et al., 2002; d'Avella and Bizzi, 2005) can be described by a low-dimensional space thought to reflect synergies. In these experiments, electromyography (EMG) is measured during a variety of tasks and matrix factorization algorithms, such as nonnegative matrix factorization (NNMF), are used to determine a subset of vectors, or synergies, which describe the EMG signals. For postural control, gait, and upper-extremity tasks in humans, a lower dimensional space of four to six synergies have consistently been shown to describe muscle activity (Ivanenko et al., 2006; Torres-Oviedo et al., 2006; Roh et al., 2012).

Using NNMF or other algorithms to identify synergies relies upon measuring EMG from the muscles used to execute a task. However, due to constraints on time, resources, and subject comfort, EMG can usually only be measured for a subset of the muscles involved in the task. For example, in the human arm, there are over twenty muscles that may contribute to movement and force generation. Previous studies of synergies in the human arm typically only measure EMG from eight to nineteen muscles (d'Avella et al., 2006; Cheung et al., 2009; Roh et al., 2012). Researchers typically try to include as many muscles as possible within the constraints of their experimental set-up and often choose larger muscles thought to contribute to the task and from which it is easier to record surface EMGs. However, no rigorous analysis has been performed to determine how the number and choice of muscles included affects the results of synergy analyses.

A few studies have been able to measure EMG from all muscles involved in a task such as Valero-Cuevas et al. (2009) who evaluated human index finger force generation. This study determined that when EMG was measured from all muscles involved in the task, more synergies were required to describe the variance in EMG activity and a lower percentage of variance in EMG activity was explained by the synergies compared to prior studies that included EMG for a subset of muscles. Furthermore, studies that have tried to use synergies to drive musculoskeletal simulations have found that the synergies identified from EMG are often inadequate for controlling motion. For example, an experimental analysis of synergies during gait did not include EMG of the iliopsoas, a deep muscle that is difficult to measure with EMG. Simulations indicated that a synergy that included the iliopsoas was required to control gait (Neptune et al., 2009). However, even from this analysis it was not possible to determine if the number of synergies was incorrect (i.e., an extra synergy for the iliopsoas was required) or the structure of the synergies was incorrect (i.e., the iliopsoas should have been included in one of the original synergies). These results suggest that synergies identified from a subset of muscles involved in a task may be inaccurate or incomplete. Understanding the sensitivity of synergy analyses to the number and choice of muscles will enable researchers to understand the limitations of these methods, design experimental protocols, and critically analyze the results of these analyses.

The aim of this study was to evaluate how the number and choice of muscles included in synergy analyses impact synergies calculated from EMG. We created a musculoskeletal model of an isometric force task in the upper-extremity and evaluated the effect of using different combinations of muscles to calculate synergies. By comparing the similarity of synergies from different combinations of muscles, we determined that synergies were affected by the number and choice of muscles included in the analysis. We also were able to identify methods, such as selecting the largest muscles, which can be used to design experimental protocols and decrease the sensitivity of synergy analyses to the number and choice of muscles.

## Materials and methods

### Musculoskeletal model

A previously developed model of the upper-extremity (Holzbaur et al., 2005) with 30 muscles (Table 1) was used to recreate an upper-extremity isometric force task that has previously been used to evaluate synergies (Roh et al., 2012). The upper-extremity model included seven degrees of freedom including three degrees of freedom at the shoulder (flexion/extension, abduction/adduction, and internal/external rotation), wrist flexion/extension, forearm supination/pronation, and two degrees of freedom at the wrist (flexion/extension and radial/ulnar deviation). The model was positioned according to the experimental protocol of Roh et al. (2012) with the hand at half an arms-length in front of the shoulder, the shoulder and elbow flexed, and the forearm and wrist in neutral positions. Muscle activations required to generate isometric forces in various directions at the hand were estimated by minimizing the sum of squared activations. Similar to the experimental protocol, the muscle activations to hold the force were examined and the periods ramping up or down from each force target were not included in the analysis. In the experimental protocol, the subjects generated forces in 54 or 210 directions evenly distributed in a sphere around the hand. Since, with a musculoskeletal model, we did not have the constraints of time, attention, or fatigue of the subject, we included 1000 force directions randomly distributed in a sphere around the hand. For each force direction, we solved for the muscle activations required to generate a ten newton force. The musculoskeletal model and analysis were executed using OpenSim, an open source software platform for musculoskeletal modeling and simulation (Delp et al., 2007).

**Table 1:** **Musculotendon actuators included in model**.

**Muscle**	**Description**	Fmax (*N*)^*^
DELT1	Anterior deltoid	1142.6
DELT2	Medial deltoid	1142.6
DELT3	Posterior deltoid	259.9
SUPSP	Supraspinatus	487.8
INFSP	Infraspinatus	1210.8
SUBSC	Subscapularis	1377.8
TMIN	Teres minor	354.3
TMAJ	Teres major	425.4
PT	Pronator teres	566.2
PECM1	Pectoralis major clavicular	364.4
PECM2	Pectoralis major medial	515.4
PECM3	Pectoralis major inferior	390.5
LAT1	Latissimus dorsi superior	389.1
LAT2	Latissimus dorsi medial	389.1
LAT3	Latissimus dorsi inferior	281.7
CORB	Coracobrachialis	242.5
TRIlong	Triceps long head	798.5
TRIlat	Triceps lateral head	624.3
TRImed	Triceps medial head	624.3
ANC	Anconeus	350.0
SUP	Supinator	476.0
BIClong	Biceps long head	624.3
BICshort	Biceps short head	435.6
BRA	Brachialis	987.3
BRD	Brachioradialis	261.3
ECRL	Extensor carpi radialis longus	304.9
ECRB	Extensor carpi radialis brevis	100.5
ECU	Extensor carpi ulnaris	93.2
FCR	Flexor carpi radialis	74.0
FCU	Flexor carpi ulnaris	128.9

* Maximum isometric force (Holzbaur et al., 2005)

### Synergy analysis

The muscle activations estimated from the musculoskeletal model during the upper-extremity isometric force task were used to calculate synergies. The muscle activations from all force directions were combined into an *m* × *t* matrix, V, where *m* was the number of muscles (i.e., 30) and *t* was the number of force directions (i.e., 1000). The activations for each muscle were normalized to unit-variance to ensure that the synergies were not biased toward high-variance muscles (Roh et al., 2012). NNMF was used to calculate synergies (Lee and Seung, 1999; Tresch et al., 1999, 2006) such that V = W^*^C where W is the *m* × *n* matrix with *n* synergies and C is the *n* × *t* matrix of synergy activation coefficients. Thus, each column of W represents the weights of each muscle for one synergy, and each row of C represents how much the corresponding synergy was activated or used to generate force in each direction. The number of synergies, *n*, was set at four to compare to the prior experimental study. The NNMF algorithm was implemented within an iterative optimization which tested random initial estimates of W and C and selected the muscle weights and activation timings that minimized the sum of squared error between V and the muscle activations.

To demonstrate that our simulation was consistent with experimental observation, we first compared the synergies estimated from the musculoskeletal model to the synergies from the experimental protocol reported by Roh et al. (2012). The experimental protocol included EMG from eight muscles: the brachioradialis, biceps brachii, triceps brachii (long and lateral heads), deltoid (anterior, medial, and posterior fibers), and pectoralis major (clavicular fibers). Thus, for this comparison, we used the activations from the musculoskeletal model for the eight muscles with EMG to calculate synergies using NNMF. We compared the synergies from the musculoskeletal model to the experimental synergies from eight unimpaired subjects. We calculated the similarity of the synergies as the average correlation coefficient. To evaluate if the synergies from the simulation were within the inter-subject variability, we compared the synergies from the musculoskeletal model to the experimental synergies of each subject. We calculated the similarity of the experimental synergies from each subject to one another to evaluate the inter-subject variability. Each subject's synergies were then compared to the simulated synergies to evaluate the similarity between the experimental and simulated synergies. We used an equivalence test to determine if the similarity of the experimental and simulated synergies were within the inter-subject similarity with a significance level of 0.05. For both the inter-subject similarity and similarity between experimental and simulated, we report the 95% confidence intervals.

### Impact of number of muscles on synergies

**Figure 1:**
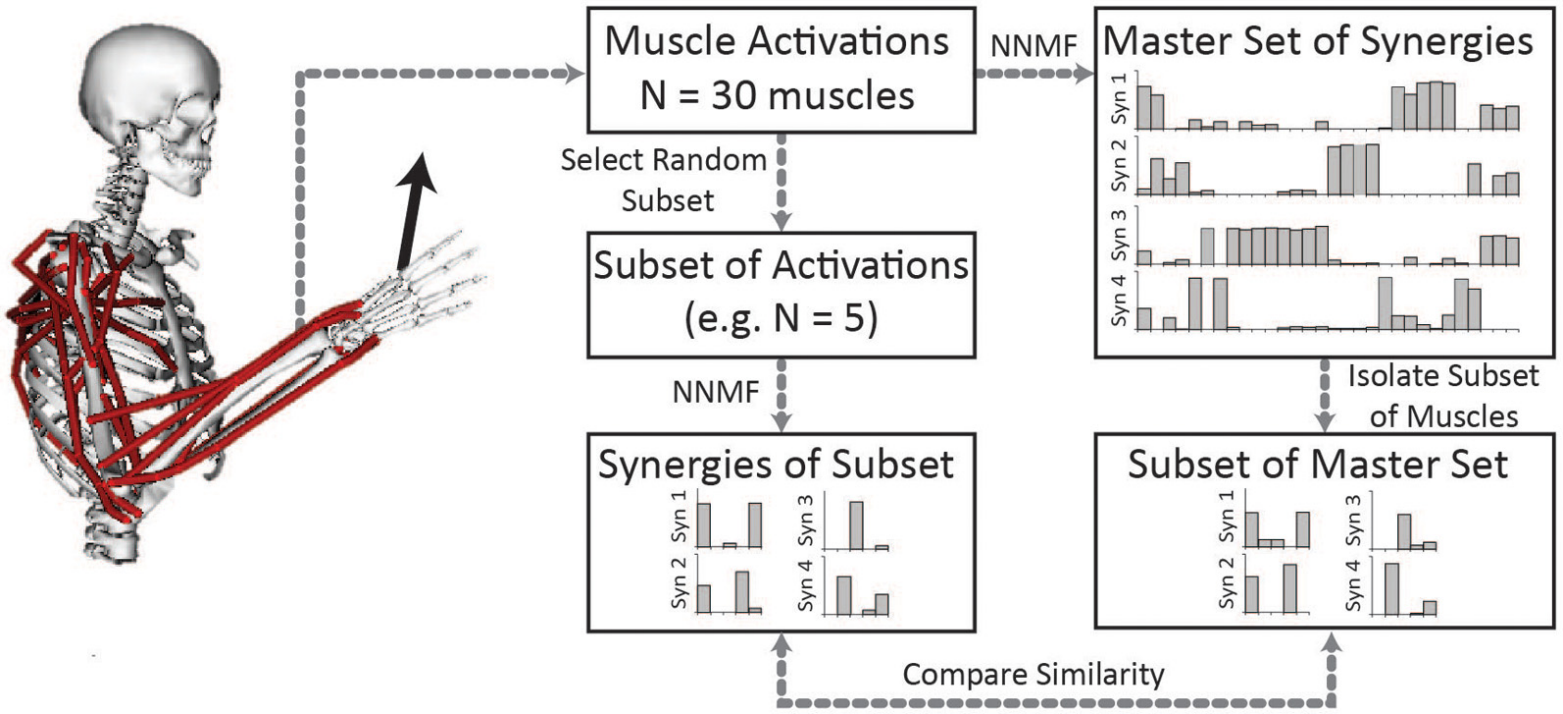
**Synergies calculated from a subset of muscles were compared to the master set of synergies calculated from all 30 muscles.** The master set of synergies was calculated using NNMF from the activations of all 30 muscles required to perform the isometric upper-extremity force task. Random subsets of muscles (varying between 5 and 29 muscles) were then selected and four synergies were calculated from the subset of muscle activations. The same subset of muscles was isolated from the master set and the similarity of the synergies was compared as the average correlation coefficient.

To evaluate the impact of the number of muscles included in the analysis on the resultant synergies, we compared the synergies calculated from random subsets of muscles to the “master set” of synergies (Figure 1). The master set of synergies was determined from the activations of all 30 muscles and 1000 force directions using NNMF. We then calculated synergies from the muscle activations of random subsets of muscles. We evaluated subsets that included odd numbers of muscles between 5 and 29. For each number of muscles (e.g., five muscles), we selected 1000 random combinations, or as many combinations possible given 30 muscles, and calculated synergies from the activations of these muscles using NNMF. The synergies from each subset were then compared to the same subset of muscles isolated from the master set. For each combination, the synergies were normalized to unit length and similarity was evaluated as the average correlation coefficient between the subset of the master set and the synergies calculated from the subset of muscle activations. The average correlation coefficient was determined by matching the pairs of synergies from the master set and subset that had the greatest similarity and averaging the correlation coefficients across the pairs. The correlation coefficient was normalized from zero to one where zero is the similarity expected by chance and one is perfect similarity (Tresch et al., 2006). The similarity expected by chance was calculated as the average correlation coefficient comparing each set of synergies to 25 randomly generated sets of synergies of the same size (i.e., same number of muscles and force directions). Across all analyses, four synergies were calculated from NNMF.

To evaluate the impact of changes in synergy weights, W, on the recruitment of synergies, C, we compared the directional tuning of synergies. The directional tuning was calculated as the dot product of the activation level of each synergy and each force direction. The resulting direction indicates the force direction for which a given synergy was highly recruited. The impact of the number of muscles on synergy recruitment was evaluated as the average angle between the directional tuning of the synergies from each subset of muscles and the master set.

Experimental EMG is commonly a noisy signal. To evaluate the impact of noise on synergies we repeated the analysis for each number of muscles between 5 and 29 with varying levels of noise. Noise was added to the estimated activations as a random normal distribution with a signal-to-noise ratio (SNR) between 0 and 20 dB, adjusted for the level of activity in each muscle. The similarity of the estimated synergies with noise was compared to the master set, as described above.

### Impact of the choice of muscles on synergies

To improve the similarity of synergies estimated from a subset of muscles to the master set, we evaluated different methods for choosing which muscles to include in the analysis. We evaluated two protocols for choosing muscles and compared the protocols to randomly selected subsets of muscles. We first evaluated the impact of selecting random sets of muscles from the dominant muscles of the master set of synergies. Dominant muscles were defined as muscles whose weight was within twenty percent of the maximum weight for each synergy, of which 22 of the 30 muscles met this criterion in at least one synergy. We varied the threshold for defining dominant muscles from 5 to 30% and found similar results for the similarity. To select a subset of muscles from the group of dominant muscles, an equal number of muscles were chosen from the dominant muscles of each synergy. For example, for combinations of five muscles, one dominant muscle was selected from each of the four synergies and then the final muscle was randomly selected from all the remaining dominant muscles. We compared the similarity of random combinations of 5–21 muscles selected from the dominant muscles to the similarity of the random combinations of muscles described above.

Selecting dominant muscles based on a synergy analysis of all relevant muscles requires that researchers have access to a musculoskeletal model appropriate for simulating their experimental protocol. For cases when this requirement may not be practical, we also evaluated the impact of selecting muscles according to size. We selected the largest muscles, according to maximum isometric force (Table 1), and determined the similarity to the master set for subsets that included from 5 to 29 of the largest muscles. These methods were evaluated to provide guidance for experimental protocols and to assist researchers in deciding which muscles to measure EMG from for synergy analyses.

## Results

### Comparison of experimental and model synergies

**Figure 2:**
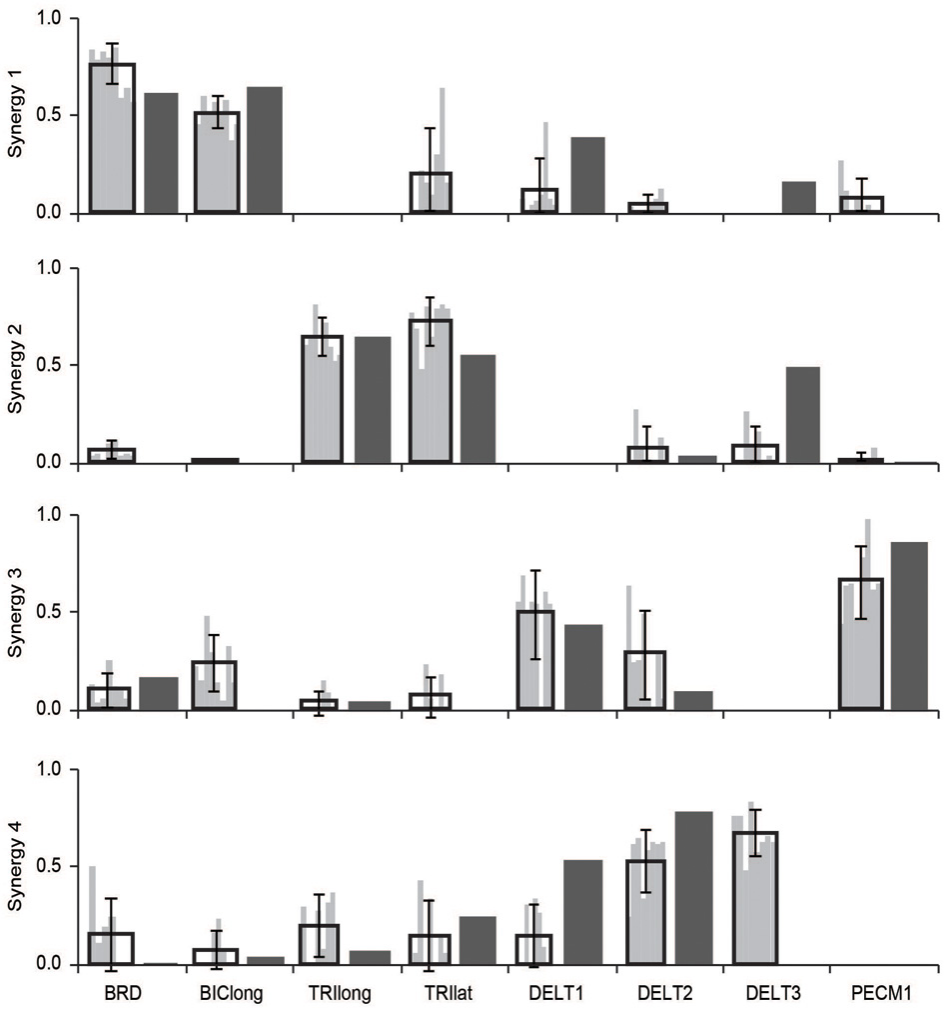
**Comparison of synergies calculated from the musculoskeletal model (dark gray bars) and experimental EMG (black outlined bars showing average ± one standard deviation and light gray bars showing synergies of individual subjects).** The similarity of the synergies from the musculoskeletal model and the experimental EMG were not significantly different from the inter-subject similarity of synergies.

Synergies estimated from a musculoskeletal model of an upper-extremity isometric force task were similar to synergies calculated from experimental EMG (Figure 2). From the experimental EMG, the average similarity of synergies between subjects was 0.79 ± 0.10 (95% CI: 0.75–0.82). We also calculated synergies from the musculoskeletal model using the activations of the eight muscles that had EMG in the experimental protocol. The average similarity of the synergies from the musculoskeletal model to the eight subjects' experimental EMG was 0.72 ± 0.10 (95% CI: 0.65–0.78). Thus, the similarity of synergies estimated from the musculoskeletal model to the experimental synergies was slightly less than the inter-subject similarity of synergies from experimental EMG, but not significantly different. From Figure 2, the primary difference between the model and experimental synergies was the grouping of the posterior deltoid. In the synergies from the model, the posterior deltoid (DELT3) was grouped with the triceps while, in the synergies from the experimental EMG, the posterior deltoid was grouped with the other compartments of the deltoid. This may be due to the simplified shoulder (i.e., ball and socket) used in the model which does not include the posterior deltoid's role in controlling other shoulder degrees of freedom.

### Synergies calculated from all muscles

**Figure 3:**
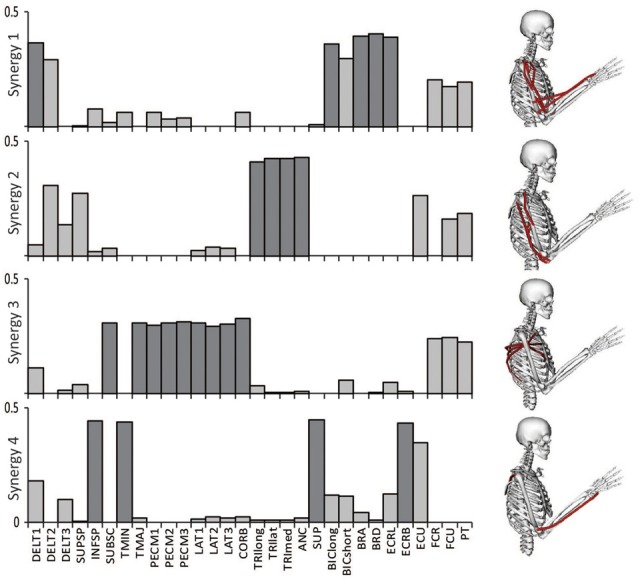
**Synergies calculated from all 30 muscles included in the model during an upper-extremity isometric force task.** The dominant muscles of each synergy (defined as within 20% of the maximum value of each synergy) are shown in dark gray and on the musculoskeletal models.

**Figure 4:**
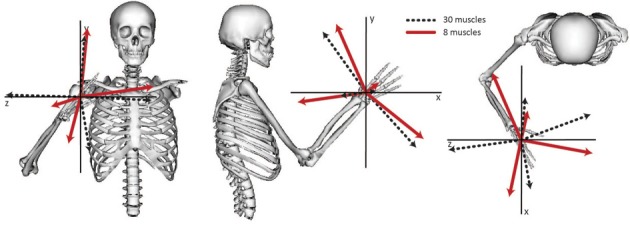
**Directional tuning of the four synergies calculated from all 30 muscles and the subset of eight muscles included in the experimental analysis.** The direction for each synergy was calculated from the activation level of each synergy across all force directions and normalized to unit length.

The relative weightings and grouping of muscles in the synergies calculated from all 30 muscles differed from the synergies calculated from the eight muscles included in the experimental protocol (Figure 3). When all 30 muscles were included in the analysis, four synergies described 88% of the total variance in muscle activity. Similar to the analysis with eight muscles, one synergy was dominated by the biceps (Figure 3, see Synergy 1) and another synergy was dominated by the triceps (see Synergy 2); however, the dominant muscles of the other synergies included muscles that were not included in the experimental analysis such as the latissimus muscles (see Synergy 3) and shoulder rotator cuff muscles and forearm muscles (see Synergy 4). Additionally, the grouping and relative weights of muscles differed with thirty versus eight muscles. For example, in the analysis with eight muscles, the deltoids dominated Synergy 4 and were also coupled with the pectoralis major clavicular (PECM1) in Synergy 3. However, when all 30 muscles were included in the analysis, the deltoids did not dominate one of the synergies and were no longer coupled with PECM1, suggesting fundamental differences in the grouping of muscles. The changes in the synergy weights, W, also altered the synergy activations in the C matrix. The directional tunings of synergies calculated from all 30 muscles were different from synergies calculated from the subset of eight muscles used experimentally, with differences in direction ranging from 12.2° to 74.5° (Figure 4).

If 5 synergies were included in the analysis, the variance accounted for increased to 92% and included four synergies with the same dominant muscles as the analysis with four synergies. The dominant muscles of the fifth synergy included the posterior deltoid and supraspinatus. To maintain consistency with the prior experimental analysis, four synergies were used for all subsequent analyses.

### The impact of number of muscles on synergies

**Figure 5:**
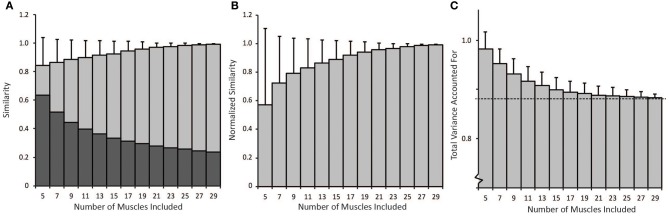
**(A)** Non-normalized similarity calculated as the average correlation coefficients of synergies calculated from random subsets that included between 5 and 29 muscles to the synergies calculated from all 30 muscles (light gray bars − average ± 1 standard deviation). The dark gray bars show the similarity expected by chance for each number of muscles included in the analysis. **(B)** Average similarity of synergies from random subsets to synergies calculated from all 30 muscles normalized by similarity expected by chance. **(C)** Average total variance accounted for by synergies from random subsets. As the number of muscles in the analysis increased, the total variance accounted for approached the variance accounted for when all 30 muscles were included in the analysis (dotted line).

The number of muscles included in the synergy analysis impacted the results from the NNMF algorithm (Figure 5). We compared the similarity of the synergies calculated from random subsets that included between 5 and 29 muscles to the synergies calculated from all 30 muscles (the master set). The average similarity of the random subsets to the master set was greater than 0.8 for all subsets that included between 5 and 29 muscles (Figure 5A). However, the similarity expected by chance increased when fewer muscles were included in the analysis. When only five muscles were included in the analysis, the similarity expected by chance was 0.63 (see dark bars, Figure 5A). Thus, the average normalized similarity (with 0 equal to similarity expected by chance) was only 0.57 ± 0.54 with five muscles (Figure 5B) and remained below 0.8 when less than 11 muscles were included in the analysis. When a small number of muscles were included in the analysis, the variance in similarity was also greater between subsets. For example, with five muscles, the average normalized similarity was 0.57 but some combinations of muscles approached perfect similarity while the similarity of other combinations was not different from similarity expected by chance. The set of five muscles with the greatest normalized similarity (0.999) included the triceps lateral head, teres major, supinator, latissimus dorsi inferior, and brachioradialis. The normalized similarity of the subset of eight muscles used in the experimental protocol to the master set of synergies was 0.52.

The difference in directional tunings of the synergy activations (C matrix) between subsets of muscles and the master set decreased as more muscles were included in the analysis. The average difference in directional tuning compared to the master set of synergies was 26.2° (±15.3°) when only five muscles were included; however, the difference in directional tuning decreased to 12.7° (±11.0) when 15 muscles were included and approached zero degrees as the number of muscles increased. Differences in directional tuning would indicate errors in interpreting how synergies are recruited to produce force in various directions.

Total variance accounted for is often used to determine the number of synergies to include in an analysis and to evaluate how well a set of synergies reproduces muscle activity. The average total variance accounted for decreased with the number of muscles included in the analysis (Figure 5C). Thus, the average total variance accounted for was 0.98 ± 0.03 when only five muscles were included in the analysis and decreased to approach the total variance accounted for by the master set of synergies, 0.88 (see dotted line, Figure 5C) as more muscles were included in the analysis. Four synergies could more easily describe the variability in muscle activity when fewer muscles were included in the analysis. These results demonstrate that experimental analyses that include fewer muscles may over-estimate the total variance accounted for compared to an analysis that included all muscles involved in a task.

The task simulated in this analysis included 1000 force directions; however, the experimental protocol included either 54 or 210 force directions. We evaluated the impact of the number of force directions on the normalized similarity and total variance accounted for. The total variance accounted for was not sensitive to the number of force directions; however, the normalized similarity was reduced when fewer than 100 force directions were included. Additionally, there was greater variability in the normalized similarity between trials when fewer force directions were included depending upon the choice and dispersion of the force directions.

### The impact of noise on synergies

**Figure 6:**
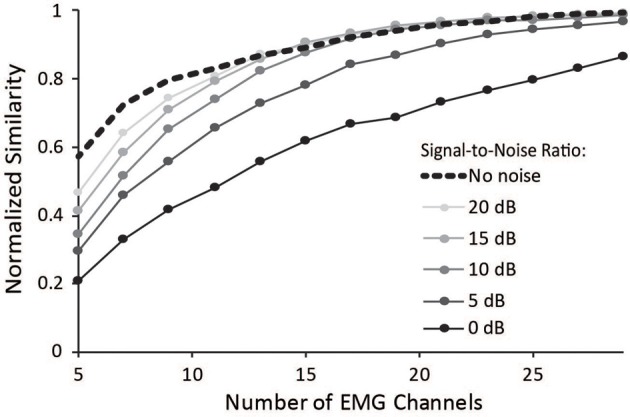
**Average normalized similarity vs. number of muscles included in the synergy analysis for varying levels of noise.** Noise was specified according to a signal-to-noise ratio between 0 and 20 dB.

Surface EMG is an inherently noisy signal; however, the activations from a musculoskeletal model estimate muscle activity without noise. Thus, we sought to determine the impact of noise on the similarity of synergies to the master set of synergies. Increasing noise decreased the average normalized similarity (Figure 6), especially for combinations that included less than 15 muscles. Noise with an SNR greater than 10 dB had minimal effect on combinations of muscles that included more than 15 muscles. These results emphasize the importance of maintaining high SNR, especially when fewer muscles are included in the analysis.

### Protocols to improve similarity of synergies

**Figure 7:**
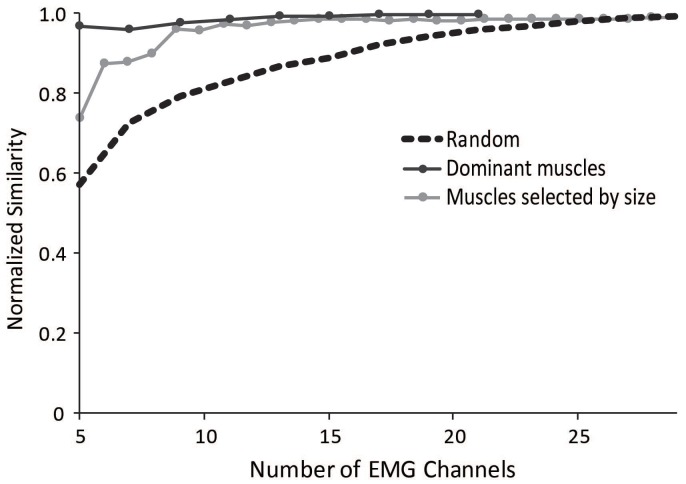
**Average normalized similarity of subsets of muscles chosen randomly (dotted black line), muscles chosen from subsets of dominant muscles from the master set (dark gray line), and muscles selected by size starting with the largest muscles (light gray line)**.

To aid researchers in selecting muscles to include in an experimental protocol for synergy analyses, we evaluated several methods that could minimize the impact of measuring EMG from a subset of muscles. The most successful method involved selecting a subset of muscles evenly distributed across the dominant muscles from the master set of synergies (Figure 7). The dominant muscles were defined as muscles that were within 20% of the maximum for each synergy; however, the effectiveness of this method remained similar if the cut-off for defining dominant muscles was varied between 5 and 30%. This protocol resulted in a normalized similarity to the master set greater than 0.95 for subsets including between 5 and 29 muscles.

We also evaluated a method in which the subset of muscles was selected based on muscle size, using the maximal isometric force of each muscle. Such a method might be useful for the general situation where a musculoskeletal model is not available but overall muscle sizes might be. Selecting the largest muscles significantly improved the similarity to the master set. The normalized similarity of synergies calculated from the five largest muscles was 0.75 and all combinations with more than seven muscles had an average normalized similarity greater than 0.9.

## Discussion

In this study we sought to determine if the number and choice of muscles with EMG in an experimental protocol will impact the synergies identified using matrix factorization algorithms, such as NNMF. We found that the number and choice of muscles does impact the structure of synergies and the amount of variance in muscle activity accounted for by a given set of synergies. However, we were also able to identify several strategies that can be used to minimize the impact of using a subset of muscles. We also compared our results from the musculoskeletal model to experimental results and found similar synergies, suggesting that results from musculoskeletal modeling were comparable to experimental conditions and can provide a platform for investigating muscle synergies.

The average similarity of synergies to the master set dropped below 0.8 when fewer than eleven muscles were included in the analysis. Prior studies of synergies during upper-extremity tasks have included between 8 and 19 muscles and thus may be significantly impacted by the choice of muscles. For example, our comparison to synergies from eight muscles used in the experimental protocol by Roh et al. (2012) had a low normalized similarity to the master set of only 0.52. Although the structure of two of the four synergies, dominated by the biceps and triceps, were similar, the other two synergies identified from the master set were dominated by muscles not included in the experimental protocol. Furthermore, the relative weighting and grouping of muscles also changed significantly when all 30 muscles were included in the analysis. Although synergies identified from a subset of muscles may be able to describe the variance in EMG activity, they may not correctly reflect how muscles are recruited or activated together which can impact the functional interpretations of synergies. Previous studies of similar tasks have also identified synergies with different structures and dominant muscles which may be due to the different muscles included in the experimental protocols (e.g., Cheung et al., 2012; Roh et al., 2013). Evaluating muscle synergies from a subset of muscles may still be valuable for comparing populations, such as unimpaired individuals and individuals after stroke, if the same subset of muscles is used for all groups. However, the limitations of using a subset of muscles involved in a task should be considered in analyzing the results of synergy analyses and in generalizing results to the over-arching neuromuscular control strategy.

Variance accounted for is also commonly used as a measure to evaluate the results of muscle synergy analyses and to determine the number of synergies used in a given task. However, the results of this study highlight that when fewer muscles are included in the analysis, the variance accounted for is over-estimated. Using variance accounted for to determine the number of synergies may result in fewer muscle synergies being selected than if all muscles were included in the analysis. The impact of the number of muscles included on variance accounted for suggests that complementary methods, such as using the ability of synergies to discriminate between tasks (Delis et al., 2013), should be used to determine the number of synergies in experimental protocols that include a small subset of muscles.

To assist with future experimental design, we identified several strategies to select muscles which can improve similarity when only a subset of muscles is included due to experimental constraints. Selecting from the dominant muscles of the synergies identified from musculoskeletal simulation was the most successful method. By selecting an equal number of dominant muscles from each synergy in the master set, the average similarity increased to over 0.95, even for cases with only five muscles. This approach worked well because it identified important muscles from each synergy of the master set which translated to similar synergies identified from NNMF. However, since musculoskeletal simulation may not always be available for analysis, we determined that selecting the largest muscles, as determined by maximum isometric force, also improved similarity. The largest muscles have the greatest contribution to movement and force generation and overlap with the dominant muscles identified with musculoskeletal simulation. These strategies for choosing which muscles to include in an experimental protocol are important for decreasing the sensitivity of synergies to experimental constraints and improving our understanding of the generalizability of synergy analyses.

The synergies calculated from the musculoskeletal model were similar to the experimental synergies; however, it is important to note that the muscle activations from the model were determined without reference to synergies. Similar to previous studies, we determined the muscle activations required to perform the task by minimizing the sum of squared activations. Thus, no synergies or other coupling between muscles was incorporated into the musculoskeletal model. Previous studies have suggested that the lower-dimensional subspace determined from matrix factorization algorithms may be more reflective of biomechanical or task constraints rather than the underlying neuromuscular control strategy (Valero-Cuevas et al., 2009; Kutch and Valero-Cuevas, 2012; Burkholder and Van Antwerp, 2013). The similarity between the synergies calculated from the model and experiment in this study further reinforce this theory since we could recover similar synergies as the experimental task in the absence of a control strategy based upon synergies.

This study also demonstrated how musculoskeletal simulation can be used to complement and optimize experimental design for muscle synergy analyses. Based upon the posture, kinematics, and external forces for an experimental protocol, musculoskeletal simulation can be used to estimate expected muscle forces and test a priori the impact of experimental constraints such as the number of muscles with EMG. Musculoskeletal simulation can also be used to predict the functional impacts of altered synergies or test if synergies identified from matrix factorization algorithms can control movement (Neptune et al., 2009; Allen and Neptune, 2012). Free musculoskeletal simulation platforms such as OpenSim (Delp et al., 2007) provide a variety of human and animal models as well as the simulation algorithms that can be used for these analyses.

Matrix factorization algorithms provide a valuable tool for evaluating neuromuscular control of movement through the framework of synergies. Synergy analyses can provide insight into lower-dimensional subspaces that describe muscle activity during a variety of tasks and may reflect the underlying strategies for controlling the complexities of the neuromuscular and musculoskeletal systems. Muscle synergy analyses are increasingly being used to evaluate altered neuromuscular control in clinical populations, such as individuals after stroke (Clark et al., 2010; Cheung et al., 2012; Roh et al., 2013). As the applications of muscle synergy analysis reach the clinical realm, it is even more important to understand the limitations and generalizability of these methods. Understanding if the structure of synergies is altered in clinical populations because of different control strategies, altered biomechanics, or other factors will be critical for using synergy analyses to improve treatment and will require careful experimental design. This study has demonstrated that, although synergies estimated from NNMF are sensitive to the number and choice of muscles, there are multiple strategies that can be employed to improve experimental design and decrease the sensitivity of these analyses to experimental constraints. Researchers should especially note the increased risk of over-estimating the variance accounted for by synergies when fewer muscles are used in an experimental analysis. Combining simulation and experimental studies provides a complementary platform to address these challenges and continue to refine our knowledge of how humans control movement and interact with the world.

### Conflict of interest statement

The authors declare that the research was conducted in the absence of any commercial or financial relationships that could be construed as a potential conflict of interest.
